# ^1^H, ^13^C, and ^15^N NMR chemical shift assignment of the complex formed by the first EPEC EspF repeat and N-WASP GTPase binding domain

**DOI:** 10.1007/s12104-021-10008-9

**Published:** 2021-01-21

**Authors:** Mikael Karjalainen, Maarit Hellman, Helena Tossavainen, Perttu Permi

**Affiliations:** 1 Department of Chemistry, Nanoscience Center, University of Jyvaskyla, Jyvaskyla, Finland; 2 Department of Biological and Environmental Science, University of Jyvaskyla, Jyvaskyla, Finland

**Keywords:** EPEC EspF, Intrinsically disordered protein, N-WASP, Resonance assignments, Solution NMR, Type III secretion system

## Abstract

LEE-encoded effector EspF (EspF) is an effector protein part of enteropathogenic *Escherichia coli*’s (EPEC’s) arsenal for intestinal infection. This intrinsically disordered protein contains three highly conserved repeats which together compose over half of the protein’s complete amino acid sequence. EPEC uses EspF to hijack host proteins in order to promote infection. In the attack EspF is translocated, together with other effector proteins, to host cell via type III secretion system. Inside host EspF stimulates actin polymerization by interacting with Neural Wiskott-Aldrich syndrome protein (N-WASP), a regulator in actin polymerization machinery. It is presumed that EspF acts by disrupting the autoinhibitory state of N-WASP GTPase binding domain. In this NMR spectroscopy study, we report the ^1^H, ^13^C, and ^15^N resonance assignments for the complex formed by the first 47-residue repeat of EspF and N-WASP GTPase binding domain. These near-complete resonance assignments provide the basis for further studies which aim to characterize structure, interactions, and dynamics between these two proteins in solution.

## Biological context

An intrinsically disordered protein (IDP) is a functional polypeptide which cannot fold spontaneously into a stable three-dimensional conformation and extensive disorder is important for its function. Similar segments within a protein are called intrinsically disordered regions. IDPs are often described as important components of the cellular signalling machinery, especially in eukaryotes’ proteome where IDPs are abundant (van der Lee et al. 2014; Wright and Dyson 2015). Previous research has revealed that pathogens can produce protein mimics which target the components of the cellular signalling machinery. These mimics outcompete their model proteins and change the host metabolism making it favourable to the pathogen (Davey et al. 2011; Aitio et al. 2012; Tossavainen et al. 2016; Sámano-Sánchez and Gibson 2020).

**Fig. 1:**
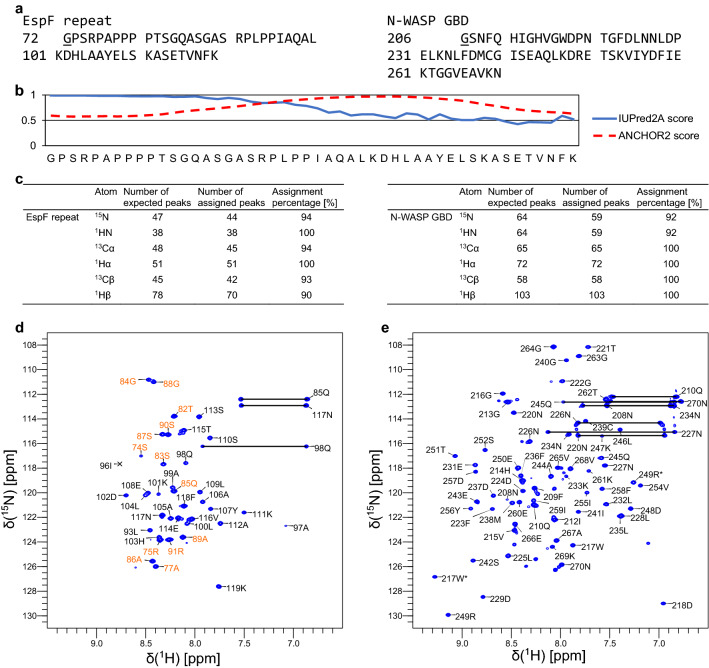
**a** The amino acid sequences of EspF and N-WASP GBD used in this study. Underlined N-terminal glycine is not part of the native sequence. **b** Predicted disorder and disordered binding regions for EspF from IUPred2A and ANCHOR2. Both programs give a score between 0 and 1 to each residue which represents the probability for disorder (IUPred2A) or the probability for locating in a disordered binding region (ANCHOR2). A higher number corresponds to a higher probability. **c** Extent of backbone assignments of EspF and N-WASP GBD. **d** Assigned ^1^H, ^15^N-HSQC spectrum of ^15^N and ^13^C labelled EspF bound to unlabelled N-WASP. The shown contour level does not display 96I, but its peak is clearly visible at lower levels. The N-terminal half amide peaks are coloured orange to point out narrow ^1^H dispersion. **e** Assigned ^1^H, ^15^N-HSQC spectrum of ^15^N and ^13^C labelled N-WASP GBD bound to unlabelled EspF. Spectra were recorded at 800 MHz and 25 °C. The NH resonances are labelled with residue numbers and single letter amino acid codes. Asparagine δ and glutamine ε side chain resonances are connected by lines. Arginine and tryptophan side chain resonances are marked by asterisk

LEE-encoded effector EspF (EspF) is one example of such a mimic, which is part of enteropathogenic *Escherichia coli*’s (EPEC’s) arsenal for intestinal infection. EspF is recognized as a type III effector protein and it is translocated from bacteria into host cells through a dedicated protein translocation apparatus, the type III secretion system. As a translocated effector, EspF interacts with cellular proteins and modifies their activity. EspF is also found in enterohaemorrhagic *E. coli* (EHEC) and *Citrobacter rodentium*. EPEC EspF is a 21 kDa IDP which contains three similar 40–47 amino acid proline-rich repeats at its C-terminus (McNamara and Donnenberg 1998; Alto et al. 2007). In this study, the first repeat (residues 73–119) was studied (Fig. 1a).

EspF repeats include amino acid sequences which mimic *bona fide* interaction sites in human proteins. The N-terminal proline-rich region has been shown to interact with the Src homology 3 (SH3) domain of sorting nexin 9 (SNX9). SNX9 is involved in clathrin-mediated endocytosis (Bendris and Schmid 2017). In the C-terminal half resides a putative Neural Wiskott-Aldrich syndrome protein (N-WASP) GTPase binding domain (GBD) binding motif (Marchès et al. 2006; Alto et al. 2007). N-WASP plays a role in actin polymerization machinery where it regulates actin filament branching and assembly through the activation of actin-related protein 2/3 (ARP2/3) complex. Alone in basal conditions N-WASP’s own autoinhibitory element, termed the C-helix, is bound to GBD domain’s hydrophobic core, keeping N-WASP inactive. For N-WASP activation, the autoinhibitory state is disrupted by the GTPase Cdc42 or other human signalling molecules. Binding of the GTPase Cdc42 to autoinhibited N-WASP leads to the release of the C-helix and exposes it for ARP2/3 complex interaction (Miki et al. 1998; Rohatgi et al. 1999; Sallee et al. 2008). It has been demonstrated that EHEC exploits this autoinhibitory interaction to promote pathogenesis. A related effector protein EspF_U_, expressed by EHEC only, can bind to N-WASP and to another Wiskott-Aldrich syndrome family protein WASP. In both interactions, EspF_U_ mimics and outcompetes the C-helix in the hydrophobic core to activate the Wiskott-Aldrich syndrome family protein (Cheng et al. 2008; Sallee et al. 2008; Aitio et al. 2012). Because previous research has shown that EspF interacts with N-WASP, the same structural mechanism of C-helix mimicry and substitution has been implied for EspF and N-WASP (Alto et al. 2007; Garber et al. 2018).

Structures of EspF_U_ in complex with N-WASP GBD and WASP GBD have been solved (Cheng et al. 2008; Aitio et al. 2012). To our knowledge, there is no published structural data on EspF in complex with N-WASP GDB. Here we report the near-complete ^1^H, ^13^C, and ^15^N resonance assignments for the complex formed by the first EspF repeat with N-WASP GBD. These initial studies enable further studies where the aim is to characterize structure, interactions, and dynamics between these two proteins in solution.

## Methods and experiments

### Protein expression and purification

Residues 73–119 were selected to represent the first repeat from EspF protein (UniProtKB B7UM88) and gene (synthetic, GenScript Inc., USA) encoding these residues was cloned to pET15b vector (Novagen) into the NdeI and XhoI sites. The protein construct carried an N-terminal His-Tag, followed by GB1 fusion protein and TEV protease (from Tobacco Etch Virus) cleavage site which was connected to EspF. Residues 207–270 were selected to represent GBD from N-WASP protein (UniProtKB O00401) and gene (synthetic, GenScript Inc., USA) encoding these residues was cloned to pET15b vector (Novagen) into the NdeI and XhoI sites. The protein construct carried an N-terminal His-Tag.

Production and purification of EspF repeat was carried out as described before (Karjalainen et al. 2020), whereas production of N-WASP GBD was done as described in Aitio et al. 2012. In the production of EspF repeat or N-WASP GBD, protein constructs expressing BL21(DE3) cells were grown in M9 minimal medium, supplemented with 1 g/l of ^15^NH_4_Cl and 2 g/l of ^13^C-d-glucose as the sole nitrogen and carbon sources, or in LB medium for obtaining unlabelled EspF repeat or N-WASP GBD. To purify N-WASP GBD, recovered clarified supernatant of His-Tagged N-WASP GBD protein was applied to the 1-ml His GraviTrap column (GE Healthcare) according to the manufacturer’s instructions. Imidazole was used to collect the N-WASP GBD protein and imidazole was removed from eluted proteins by PD-10 (GE Healthcare) before thrombin cleavage. The cleaved N-WASP GBD was concentrated and applied into the Superdex 75 (16/60). The columns were equilibrated with 20 mM sodium phosphate, pH 6.5, 50 mM NaCl buffer. Elution fractions containing purified proteins were pooled and concentrated by Vivaspin 2 (Sartorius Stedim). The gel filtration was performed by using ÄKTA Purifier FLPC purification system (GE Healthcare).

### NMR spectroscopy

All NMR spectra of the complex formed by EspF repeat and N-WASP GBD were acquired at 298 K using a Bruker Avance III HD 800 MHz NMR spectrometer equipped with a helium cooled TCI ^1^H/^13^C/^15^N cryoprobe. For the resonance assignments of the binary complex formed by EspF repeat and N-WASP GBD two samples were used. One was composed of ^15^N, ^13^C labelled EspF repeat in complex with unlabelled N-WASP GBD, the other was composed of ^15^N, ^13^C labelled N-WASP GBD in complex with unlabelled EspF repeat. Ratios between two proteins were 1:1.2 and the labelled protein was always saturated with the unlabelled protein. Protein sample concentrations varied between 0.3 and 1.0 mM and samples were loaded into 5 mm Shigemi NMR tubes. Proteins were in 4 %/96% D_2_O/H_2_O, 20 mM sodium phosphate, 50 mM NaCl, pH 6.5 NMR buffer. Chemical shifts were referenced to external 2,2-dimethyl-2-silapentane-5-sulfonic acid (DSS). Resonance assignment was carried out with the following set of experiments: ^1^H, ^15^N HSQC, constant time ^1^H,^13^C HSQC for aliphatic and aromatic regions (Cavanagh 2007), HNCACB (Grzesiek and Bax 1992a), HN(CO)CACB (Grzesiek and Bax 1992b), HBHA(CO)NH, H(CC)(CO)NH, (H)CC(CO)NH, ^1^H, ^15^N NOESY-HSQC, ^1^H, ^13^C NOESY-HSQC for aliphatic and aromatic regions (Sattler et al. 1999), (HB)CB(CGCD)HD, (HB)CB(CGCDCE)HE (Yamazaki et al. 1993; Sattler et al. 1999), HC(C)H-COSY (Kay et al. 1993), DE-MQ-(H)CC_m_H_m_-TOCSY (Permi et al. 2004), 4D (HACA)CONCAHA (Tossavainen et al. 2020), and CON (Bermel et al. 2006). NMR data were processed with TopSpin 3.5 (Bruker Corporation) and analysed with CcpNmr Analysis 2.4.2 (Vranken et al. 2005). The missing proline assignments of the N-terminal proline-rich region of EspF repeat (residues 73, 76, and 78–80) were supplemented from assignments of the free form (unpublished data).

### Assignments and data deposition

The amino acid sequences of the first EspF repeat (hereafter EspF) and N-WASP GBD are presented in Fig. 1a. For EspF, bioinformatics tools were used to analyse the sequence and predict intrinsic disorder (IUPred2A (Mészáros et al. 2018), DISOPRED3 (Jones and Cozzetto 2015), disCop (Fan and Kurgan 2014), and PrDOS (Ishida and Kinoshita 2007)). IUPred2A prediction was selected as the representative prediction and the result shows disordered characteristics for EspF (Fig. 1b). The prediction gives a near 1 score for residues in the first half of EspF, indicating that these are very likely to reside in a disordered region. The second half of the sequence shows a significantly more ordered tendency and has values at both sides of the (dis)ordered cutoff value 0.5.

Because we are expecting a disorder-to-order transition for EspF upon binding to N-WASP GBD due to similarity to EspF_U_ (Aitio et al. 2012), an additional ANCHOR2 prediction from IUPred2A is included (Fig. 1b). The prediction gives a close to 1 score for residues from 92P to 110S, indicating high probability of being part of a disordered binding region and a disorder-to-order transition upon binding to N-WASP GBD. Observations from the ^1^H, ^15^N-HSQC spectrum of bound EspF support this idea. The peaks of residues 93L to 114E have substantially shifted and become dispersed as compared to those in the spectrum of free EspF (unpublished data). The N-terminal half amide peaks, however, remain clustered in a narrow ^1^H region (Fig. 1d). These peaks have chemical shifts matching those of free EspF, which affirms the assumption that the N-terminal part is not involved in binding of N-WASP GBD.

For both proteins, extent of backbone assignments is shown in Fig. 1c. ^1^H, ^15^N-HSQC spectra highlighting the spectral quality together with the assignments are shown in Fig. 1d for EspF and in Fig. 1e for N-WASP GBD. For EspF, ^15^N 94%, ^1^HN 100 %, ^13^Cα 94 %, and ^1^Hα 100 % complete backbone assignments were attained. ^13^Cβ 93 % and ^1^Hβ 90 % were assigned. In total, side chain assignments were 84 % complete for achievable assignments. Missing assignments for prolines were supplemented from those of free EspF (unpublished data), assigned using 3D Hα-detected experiments (Mäntylahti et al. 2010, 2011). These included ^15^N assignments for prolines 73 and 76. Also, ^15^N, ^13^C’, and ^1^Hα assignments for prolines 78–80. As said, N-terminal residues do not participate in N-WASP GDB binding and proline shifts match those of the complex form.

^15^N 92 %, ^1^HN 92 %, ^13^Cα 100%, and ^1^Hα 100% complete backbone assignments for N-WASP GBD were obtained for residues 208–210, 212–252 and 254–270. Residues 207S, 211H, and 253K are missing backbone nitrogen and proton assignments. ^13^Cβ and ^1^Hβ were assigned completely. In total, side chain assignments were 92 % complete for achievable assignments. Both proteins had an N-terminal glycine residue as a cloning artefact and these are included in the reported chemical shifts.

In this manuscript, we have reported nearly complete ^1^H, ^13^C, and ^15^N resonance assignments for the complex formed by the first EspF repeat with N-WASP GBD. These near-complete resonance assignments can be used in further studies where aim is to characterize structure, interactions, and dynamics between these two proteins in solution. The assigned ^1^H, ^13^C, and ^15^N chemical shifts have been deposited in the BioMagResBank (http://www.bmrb.wisc.edu/) database with the accession number 50548.
